# Network pharmacology and experimental validation to elucidate the pharmacological mechanisms of Bushen Huashi decoction against kidney stones

**DOI:** 10.3389/fendo.2023.1031895

**Published:** 2023-02-14

**Authors:** Haizhao Liu, Min Cao, Yutong Jin, Beitian Jia, Liming Wang, Mengxue Dong, Lu Han, Joseph Abankwah, Jianwei Liu, Tao Zhou, Baogui Chen, Yiyang Wang, Yuhong Bian

**Affiliations:** ^1^ School of Integrative Medicine, Tianjin University of Traditional Chinese Medicine, Tianjin, China; ^2^ State Key Laboratory of Component-Based Chinese Medicine, Tianjin Key Laboratory of TCM Chemistry and Analysis, Tianjin University of Traditional Chinese Medicine, Tianjin, China; ^3^ Wuqing Hospital of Traditional Chinese Medicine Affiliated with Tianjin University of Traditional Chinese Medicine, Tianjin, China

**Keywords:** Bushen Huashi decoction, kidney stone, network pharmacology, Chinese medicine formula, crystal deposition

## Abstract

**Introduction:**

Kidney stone disease (KS) is a complicated disease with an increasing global incidence. It was shown that Bushen Huashi decoction (BSHS) is a classic Chinese medicine formula that has therapeutic benefits for patients with KS. However, its pharmacological profile and mechanism of action are yet to be elucidated.

**Methods:**

The present study used a network pharmacology approach to characterize the mechanism by which BSHS affects KS. Compounds were retrieved from corresponding databases, and active compounds were selected based on their oral bioavailability (≥30) and drug-likeness index (≥0.18). BSHS potential proteins were obtained from the Traditional Chinese Medicine Systems Pharmacology (TCMSP) database, whereas KS potential genes were obtained from GeneCards and OMIM, TTD, and DisGeNET. Gene ontology and pathway enrichment analysis were used to determine potential pathways associated with genes. The ingredients of BSHS extract were identified by the ultra‐high‐performance liquid chromatography coupled with quadrupole orbitrap mass spectrometry (UHPLC-Q/Orbitrap MS). The network pharmacology analyses predicted the potential underlying action mechanisms of BSHS on KS, which were further validated experimentally in the rat model of calcium oxalate kidney stones.

**Results:**

Our study found that BSHS reduced renal crystal deposition and improved renal function in ethylene glycol(EG)+ammonium chloride(AC)-induced rats, and also reversed oxidative stress levels and inhibited renal tubular epithelial cell apoptosis in rats. BSHS upregulated protein and mRNA expression of E2, ESR1, ESR2, BCL2, NRF2, and HO-1 in EG+AC-induced rat kidney while downregulating BAX protein and mRNA expression, consistent with the network pharmacology results.

**Discussion:**

This study provides evidence that BSHS plays a critical role in anti-KS *via* regulation of E2/ESR1/2, NRF2/HO-1, and BCL2/BAX signaling pathways, indicating that BSHS is a candidate herbal drug for further investigation in treating KS.

## 1 Introduction

Kidney stone disease is a urinary system disease caused by the kidney’s abnormal accumulation of crystalline material such as calcium, oxalic acid, uric acid, and cystine (1). With a prevalence of 7% to 13% in North America, 5-9% in Europe, and 1-5% in Asia, it is one of the most common diseases affecting populations all over the world (1). Recent data from the United States showed that the prevalence of stones in the United States was 8.8%, with 10.6% and 7.1% reported for their prevalence in men and women, respectively (2). Primary hyperparathyroidism (3), obesity (4), diabetes (5, 6), and hypertension (7, 8) are some of the risk factors for kidney stone formation. Patients with kidney stones are also at high risk of hypertension (7), chronic kidney disease (CKD) (9, 10), and end-stage renal disease (ESRD) (10, 11). Kidney stones always lead to several complications, such as urinary tract obstruction, hydronephrosis, infection, local damage to the kidney, and renal dysfunction (12). The formation of kidney stones is a complex process involving urinary supersaturation, nucleation, growth, aggregation, and retention of urinary stone components within the renal tubular cells (13). Surgical treatment for the removal of kidney and ureteral stones has already achieved mature development over the past several decades (14). Currently, the most common kidney stone treatments include shock wave lithotripsy, ureteroscopic fragmentation and removal, and percutaneous nephrolithotomy (14). Although surgical therapies have greatly resolved patients’ pain, postoperative adverse effects and a high recurrence rate of stones are vexing. Therefore, scientists are focusing on exploring new targets and new drugs, which is crucial to reduce the incidence and recurrence rate of kidney stones.

Traditional Chinese medicine (TCM) has been effectively used in treating diverse diseases for a long time in China and is also traditionally used to treat kidney stones (15). Different from the single-target concept of Western medicine, TCM emphasizes the concept of the body as an organic whole. Generally, a TCM prescription is composed of several herbs. Each herb typically has multiple active compounds that simultaneously act on multi-targets. Due to the complexity of the TCM components, conventional pharmacology research methods are complex to fully elucidate the potential molecular mechanism of TCM compounds in disease treatment.

BSHS is an experienced TCM prescription of the National Famous TCM Doctor, Professor Baogui Chen, commonly used to treat kidney stones and has shown remarkable effectiveness in clinical practice. BSHS is mainly composed of eight Chinese herbs, including Lysimachiae Herba, Lygodii Spora, Plantaginis Semen, Clerodendranthus Spicatus, Cibotii Rhizoma, Dipsaci Radix, Malva verticillata seed, and Licorice. It remains relatively unclear, however, what the bioactive components of THCQ are as well as their pharmacological mechanisms. During the past few years, system biology, polypharmacology, and system biology-based network pharmacology have boomed due to the increase in biomedical data. With network pharmacology, target molecules, biological functions, and bioactive compounds can be combined to form complex interaction networks, which are precisely in line with the natural characteristics of TCM and can improve our understanding of TCM’s mechanisms of action (16). A network pharmacology approach can contribute to our understanding of the multicomponent, multitarget, and multi-pathway nature of TCM. This study aimed to decipher the mechanism of action of BSHS in suppressing kidney stones by integrating network pharmacology and pharmacological evaluation.

## 2 Materials and methods

### 2.1 Reagents and materials

All medicinal plants were provided by Wuqing Hospital of Traditional Chinese Medicine affiliated to Tianjin University of Traditional Chinese Medicine (Tianjin, China). The Paishi granule (PSG) was purchased from Nanjing Tongrentang Pharmaceutical Co., Ltd. (Nanjing, China). The Urea (BUN) Assay Kit, Creatinine (Cr) Assay kit, Calcium (Ca) Assay Kit, Alkaline phosphatase (ALP) assay kit, Magnesium (Mg) Assay Kit, Superoxide Dismutase (SOD) assay kit, and Malondialdehyde (MDA) assay kit were purchased from Nanjing Jiancheng Bioengineering Research Institute (Nanjing, China). The oxalate content detection kit and BCA Protein Assay Kit were purchased from Beijing Suolaibao Technology Co., Ltd. (Beijing, China), whiles Von Kossa dye and HE dye were purchased from Wuhan Servicebio Technology Co., Ltd. (Wuhan, China). The ELISA assay kits for estradiol (E2) were purchased from cloud-clone Corp. Wuhan (Wuhan, China). The anti-estrogen receptor alpha (ERα) antibody, anti-NRF2 antibody, anti-BCL-2 antibody, and anti-Bax antibody were purchased from Abcam (USA). The Caspase-3 Antibody and Cleaved Caspase-3 Antibody were purchased from CST (USA). The Estrogen receptor beta (ERβ) Rabbit pAb and HO-1 Rabbit pAb were purchased from Abclonal (Wuhan, China). The HPLC grade acetonitrile and formic acid was purchased from Fisher Chemicals (Fisher Scientific, Waltham, MA, USA). The purified water was purchased from Guangzhou Watsons Food and Beverage Co., Ltd (Guangzhou, China).

### 2.2 Analysis of BSHS by network pharmacology

#### 2.2.1 Screening bioactive components and action targets of BSHS

We used the TCMSP database (http://www.tcmspw.com/tcmsp.php) to search for BSHS constituent medicines (Lysimachiae Herba, Lygodii Spora, Plantaginis Semen, Dipsaci Radix, and Licorice) and their chemical and pharmacological data. The remaining herbs, such as Clerodendranthus spicatus, Cibotii Rhizoma, and Malva verticillata seed were not retrieved from the database; however, their active compounds were retrieved by reviewing the literature. As parameters for screening the compounds collected, oral bioavailability (OB) and drug-like quality (DL) were selected. The OB represents the percentage of unchanged drug that reaches the systemic circulation after oral administration. DL indexes can be used to optimize pharmacokinetic and pharmaceutical properties, such as solubility and chemical stability (17). Here, we set OB ≥ 30 and DL ≥ 0.18 as criteria to screen for biologically active compounds. TCMSP was further used to screen the targets of the active ingredients of BSHS, and Uniprot was used to correct and deduplicate the drug targets. (https://www.uniprot.org/).

#### 2.2.2 The construction of the drug-active ingredient-target interaction network

The obtained active ingredients and cross-targets were sorted using Microsoft Excel worksheet. After the data were imported into Cytoscape 3.7.2 software, a “drug-active ingredient-target” network model was constructed, in which the nodes represent herbs, ingredients, and targets, while the edges represent the relationship role among the three nodes. We calculated the ‘degree’ value according to the number of associations between each node.

#### 2.2.3 Screening of potential targets for KS

We used terms such as kidney calculus, kidney stones, Nephrolithiasis, Renal calculus, and Renal stones as keywords related to screened potential targets from Genecards (https://www.genecards.org), Therapeutic Target Database (TTD, http://bidd.nus.edu.sg/group/ttd/ttd.asp), Online Mendelian Inheritance in Man (OMIM, https://www.genecards.org), and DisGeNET (https://www.disgenet.org/home/) databases. After eliminating repetitive targets, the potential targets that correlated with KS were obtained. In order to determine the intersection between BSHS and KS targets, we drew a Venn diagram.

#### 2.2.4 Construction of the protein-protein interaction network and screening of key targets

In order to clarify the functional interactions between the screened potential proteins, we constructed a protein-protein interaction (PPI) network using the STRING database (https://string-db.org/). The PPI network was inputted into Cytoscape 3.7.2 software using the CytoNCA software to analyze the topology of the intersection network. Further, we took the nodes with the ‘degree’ value greater than twice the median as the basis for screening the key targets and finally got the critical targets of BSHS for treating kidney stone disease.

#### 2.2.5 KS-related target gene ontology and KEGG pathway enrichment analysis for BSHS

The analysis of GO enrichment and KEGG pathways was conducted by DAVID Bioinformatics Resources 6.8 (http://david.ncifcrf.gov). For functional annotation clustering, terms with thresholds of Count ≥ 2 and Expression Analysis Systematic Explorer (EASE) scores ≤ 0.05 were selected.

### 2.3 Experimental verification

#### 2.3.1 Preparation of BSHS

Eight raw herbs of BSHS were provided from the pharmacy department of the Wuqing Hospital of Traditional Chinese Medicine affiliated with Tianjin University of Traditional Chinese Medicine(Tianjin, China) (**Supplementary Figure 1**). Lysimachiae Herba, Lygodii Spora, Plantaginis Semen, Clerodendranthus Spicatus, Cibotii Rhizoma, Dipsaci Radix, Malva verticillata seed, and Licorice were mixed in proportions of 3:1.5:1.5:3:1.5:1.5:1.5:1(w/w) respectively and then soaked in 12 times the volume of distilled water (v/m) for 1 h, decocted twice, at 1.5 h per decoction. After concentrating the decoction to 1 g/mL, it was stored at -20°C until used.

#### 2.3.2 UHPLC-Q/Orbitrap MS analysis of SM ethanol extract

##### 2.3.2.1 Preparation of test solution

Weigh 20 mg of BSHS extract in a 1.5 mL centrifuge tube, add 1 mL of pure water, vortex for 2 min, sonicate for 10 min, dilute 10 times with pure water, centrifuge at 1,4000 rpm for 20 min to extract the supernatant and leave for measurement.

##### 2.3.2.2 Chromatographic conditions

The chromatographic column was a Waters ACQUITY UPLC^®^ BEH C18 column (1.8 μm, 2.1 × 100 mm); the mobile phase was 0.1% formic acid in water (A) -acetonitrile (B). Gradient elution (0-2 min, 3% B; 2-6 min, 3-23% B; 6-10 min, 23-23.5% B; 10-10.5 min, 23.5-35% B; 10.5-11 min, 35-40% B; 11-15 min, 40-45% B; 15-16 min, 45-100% B; 16-17 min, 100% B; 17.01 min, 3% B; 18 min; 3% B); Flow rate: 0.4 mL/min. Column temperature: 40°C; The injection volume was 5.0 μL.

##### 2.3.2.3 Mass spectrometry conditions

The HESI (heated electrospray ionization probe) parameters were as follows: spray voltage, -3.0 kV/+3.5 kV; sheath gas, (N2) 35 L/h; auxiliary gas (N2), 10 L/h; purge gas (N2), 0 L/h; capillary temperature, 350 °C; auxiliary gas heater temperature, 350 °C. The Full MS/dd-MS^2^ scan method is used, with simultaneous detection in both positive and negative ion modes. The MS^1^ full Scan Range was *m/z* 100-1500 with a resolution of 70000; MS^2^ mass spectrometry scan was dynamic mass range with a resolution of 17500; automatic gain control (AGC) for MS^1^ and MS^2^ were set to 3 × 10^6^ and 1 × 10^5^ respectively; maximum injection time was defined as 100 ms and 50 ms for MS^1^ and MS^2^, respectively; collision energy (HCD) was performed at a normalised collision energy (NCE) of 10/30/50 V; isolation width was set to 4.0 *m/z*; dynamic exclusion time was 10 s.

##### 2.3.2.4 Data processing

The raw mass spectrometry data files of BSHS extracts were imported into Compound Discover software for automatic identification according to the Natural Product database, followed by processing of the analytical results from Compound Discover. Xcalibur software was used to identify and characterize the chemical components of BSHS.

### 2.4 Establishment and grouping of a rat model of calcium oxalate kidney stones

Male SD rats (n=40) weighing 180-220 g were purchased from Beijing Viton Lever Laboratory Animal Technology Ltd. (Beijing, China). All animals were housed under standard laboratory conditions with free access to water and food. After a 7-day environmental adaptation period, the rats were randomly divided into four groups, i.e., the normal control group, model group, PSG group, and BSHS group, with 10 rats in each group. The rats in the normal control group were given free water and were intragastrically injected with saline (2 ml/d) after two weeks. The rats in the model group were given 0.75% EG (v/v) + 0.75% AC (w/v) free water for two weeks and were intragastrically injected with saline (2 ml/d) after two weeks. The rats in the PSG group were given 0.75% EG (v/v) + 0.75% AC (w/v) free water for two weeks and were intragastric injected with PSG (6.17 g/kg/d) after two weeks. The rats in the BSHS group were given 0.75% EG (v/v) + 0.75% AC (w/v) free water for two weeks and were intragastric injected with BSHS herbal concentrate (11.6 g/kg/d) after two weeks. A day before the execution, 24 h urine samples were collected from all the rats and stored at -80°C. On day 28, the animals were treated with anesthesia, and blood samples were taken from the abdominal aorta and centrifuged at 3500 rpm for 15 min at 4°C. The serum supernatant was aspirated and stored at -80°C. The left kidney was fixed in 4% paraformaldehyde and embedded in paraffin for hematoxylin and eosin (H&E) staining, Von Kossa staining, and TUNEL staining. After snap-freezing in liquid nitrogen, the right kidney was stored at -80°C. All animal experiments were performed according to the requirements of the Experimental Animal Ethics Committee of Tianjin University of Traditional Chinese Medicine.

### 2.5 Renal pathological examination and crystal deposition assay

The left kidney was fixed in 4% paraformaldehyde, dehydrated in gradient alcohol and embedded in paraffin as described by Qian et al (18). Longitudinal 4-µm paraffin sections were prepared for the H & E and Von Kossa staining. These sections were observed under the fluorescence microscope (Olympus IX2-UCB, Japan) to confirm the presence of crystals in the stained materials. The formed crystals were evaluated using professional image analysis software (ImageJ, U.S.A). Each section was photographed with 20 randomly selected fields of view under a 200× microscope. The calculations of the stone area were determined for each section using Image J software to obtain the sum of stone area under 20 fields of view and the percentage of stone area, i.e., $\text{percentage of stone area}=\frac{\text{stone area}}{\text{longitudinal section area of kidney}}*100$ .

### 2.6 Urine volume and renal/body weight index in rats

On day 28, we fed rats in metabolic cages and collected 24 h of urine using 0.02% sodium azide to prevent bacterial growth and recording of 24 h urine volume in rats. The kidneys were removed bilaterally, stripped of peritoneum and fat, and then weighed. $\text{Renal}/\text{body weight index}=\frac{\text{renal weight}}{\text{body weight}}*100$ .

### 2.7 Renal biochemical examination

The right kidneys were placed in ice-cold phosphate-buffered saline, pH 7.4, and homogenized using a tissue homogenizer. Commercial kits were used strictly according to the manufacturer’s instructions to determine the levels of malondialdehyde (MDA), superoxide dismutase (SOD), oxalate, and calcium (Ca) in the tissue.

### 2.8 Urine biochemical examination

On day 28, we fed rats in metabolic cages and collected 24 h of urine using 0.02% sodium azide to prevent bacterial growth. The levels of urinary oxalate, Ca, phosphorus (P), and magnesium (Mg) were examined by utilizing the commercial kits on the microplate reader (Varioskan Flash, Thermo Scientific) following the instructions of the manufacturers.

### 2.9 Serum biological parameters analysis

After 2 hours at room temperature, blood samples were centrifuged for 15 minutes at 3500 rpm (4°C). The supernatants were collected and stored at −80°C until use. The levels of serum Ca, P, Mg, creatinine (Cr), and urea nitrogen (BUN) were examined by utilizing the commercial kits in the microplate reader following the instructions of the manufacturers.

### 2.10 Terminal deoxynucleotidyl transferase dUTP nick-end labeling staining

We used a TUNEL assay kit according to the instructions of the manufacturer to assay renal apoptosis in left kidney tissues embedded in paraffin and cut into 4mm thick sections. Cells positive for TUNEL were counted in 5 randomly selected fields (400x magnification) under a fluorescence microscope (Olympus IX2-UCB, Japan). The rate of apoptotic cells was analyzed using Image J (USA).

### 2.11 ELISA assay

After 2 hours at room temperature, blood samples were centrifuged for 15 minutes at 3500 rpm (4°C). The supernatants were collected and stored at -80°C until used. The levels of E2 were measured with commercial ELISA kits following the protocols of the manufacturer.

### 2.12 Quantitative real time polymerase chain reaction

Total RNA from frozen right renal tissue was isolated using RNA simple Total RNA Kit (Tiangen, Beijing, China) and then reverse-transcribed to cDNA with a Reverse Transcription Kit (Tiangen, Beijing, China). The qPCR was performed using Bio-Rad IQ5 (Bio-Rad, USA) and according to the manufacturer’s protocols for the setup procedure. The housekeeping gene GAPDH was used for normalization. The fold changes were calculated using the method of 2^-ΔΔCt^. All primer sequences used in this study have been shown in **Table 1**.

**Table 1:** Primer sequences.

Genes	Primer sequence(5’-3’)
*β-actin*	Forward : CCTCTATGCCAACACAGTGC
	Reverse : CCTGCTTGCTGATCCACATC
*Esr1*	Forward : GCACCATCGATAAGAACCGG
	Reverse : TTCGGCCTTCCAAGTCATCT
*Esr2*	Forward : AGGATGTACCACCGAATGCCAAGT
	Reverse : TCCAAGTGGGCAAGGAGACAGAAA
*Nrf2*	Forward : GCCTTCCTCTGCTGCCATTAGTC
	Reverse : TGCCTTCAGTGTGCTTCTGGTTG
*Ho-1*	Forward : TATCGTGCTCGCATGAACACTCTG
	Reverse : GTTGAGCAGGAAGGCGGTCTTAG
*Bcl2*	Forward : CTTCAGGGATGGGGTGAACT
	Reverse : ATCAAACAGAGGTCGCATGC
*Bax*	Forward : GACGCATCCACCAAGAAGCTGAG
	Reverse : GCTGCCACACGGAAGAAGACC

### 2.13 Western blot analysis

RIPA buffer (Solarbio Co., Ltd. Beijing, China) was used to extract proteins from kidney tissues. Protein needs to be denatured by boiling it in a metal bath at 100°C for 10 minutes. The total protein concentration was determined using the BCA protein assay kit (Solarbio, Beijing, China). An equal amount of protein (20 µg) was separated using 10% sodium dodecyl sulfate-polyacrylamide gel electrophoresis (SDS-PAGE) and then electrophoretically transferred onto PVDF (Millipore. Billerica, MA, USA). The membranes were blotted with 5% fat-free milk in tris buffer saline with tween 20 (TBST) buffer for 2 h at room temperature and then incubated at 4°C overnight with primary antibodies: anti- GAPDH (1:1,0000), anti-ERα (1:1,000), anti- ERβ (1:1,000), anti- NRF2 (1:1,000), anti-HO-1 (1:1,000), anti-BCL2 (1:1,000), anti-BAX (1:1,0000), anti-Caspase-3 (1:1,000) and anti-Cleaved Caspase-3 (1:1,000). Afterward, the membranes were incubated with HRP-conjugated anti-rabbit/mouse IgG. The blots were imaged under an enhanced chemiluminescence (ECL) system. The target band molecular weights and net optical density were analyzed using the multifunctional imager (Jena UVP Chem studio, Germany).

### 2.14 Statistical analysis

All data are presented as mean values ± SD, and graphs were created and analyzed using Prism Software (GraphPad Prism 7). The one-way analysis of variance (ANOVA) was used to evaluate the differences among the groups. It was deemed statistically significant when the p<0.05.

## 3 Results

**Figure 1:**
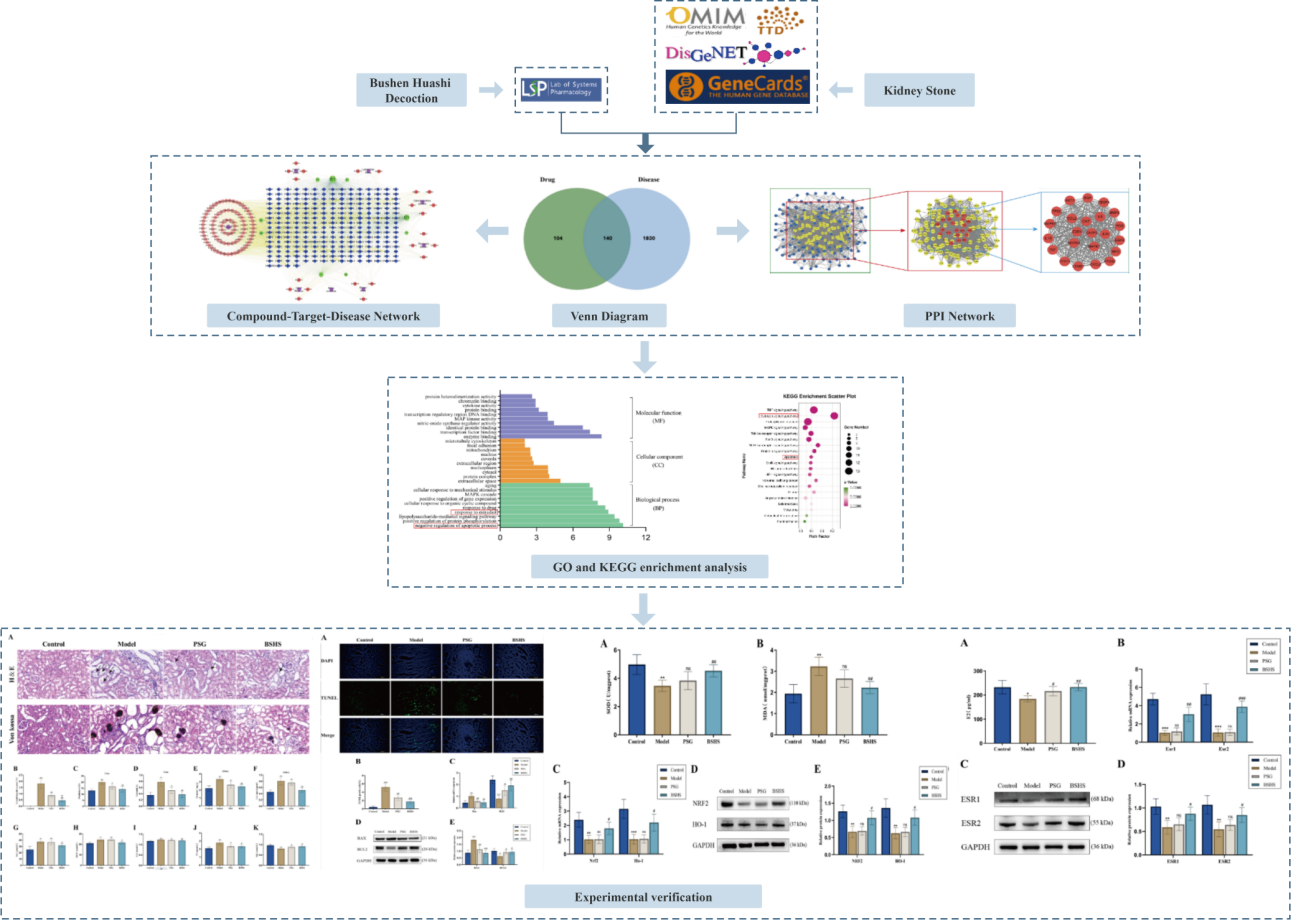
The flowchart illustrates the mechanism of BSHS in KS, from target identification, network construction, and enrichment analysis to experimental validation.

In this study, we identified BSHS-related active compounds, critical therapeutic targets, and the molecular mechanism of action of BSHS in kidney stone disease treatment by network pharmacology, functional gene pathway analysis, network analysis, and other comprehensive methods. Finally, we predicted the potential molecular mechanisms of BSHS and validated *in vivo* experiments. A flowchart of this research is shown in **Figure 1**.

### 3.1 Screening of BSHS bioactive ingredients and therapeutic targets for KS

**Figure 2:**
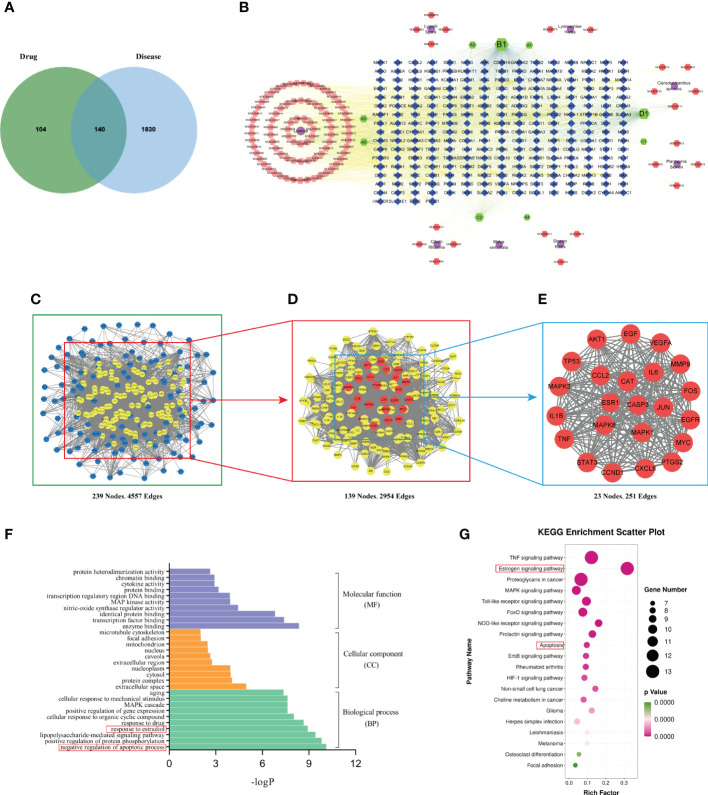
Network pharmacology analysis. **(A)** Target intersections between BSHS and KS. **(B)** The network of drug-compound-target included 8 kinds of herbs, 126 active components, and 244 target genes. Purple circle: drug, orange and green hexagon: active ingredients of BSHS; blue quadrilateral: targets. **(C)** A PPI network of predicted BSHS targets against KS. **(D)** A list of significant proteins from the PPI network was derived from **(C)**. **(E)** A list of 23 key proteins of BSHS in KS treatment was derived from **(D)**. **(F)** Based on the GO enrichment analysis, these are the top 10 indicators of BP, CC, and MF. **(G)** The top 20 signaling pathways were identified according to KEGG.

We obtained 10 active ingredients of Lysimachiae Herba, 10 active compounds of Lygodii Spora, 9 active ingredients of Plantaginis Semen, 8 active ingredients of Dipsaci Radix, 92 active ingredients of Licorice, 6 active ingredients of Clerodendranthus spicatus, 8 active ingredients of Cibotii Rhizoma, and 1 active ingredient of Malva verticillata seed. After removing duplicate entries, a total of 126 active ingredients and 244 ingredient action targets were obtained. A total of 1970 targets related to KS treatment were obtained from four databases: Genecards, OMIM, DisGeNET, and TTD. Using the online Venn diagram editing website (http://jvenn.toulouse.inra.fr/app/example.html), 140 potential target genes were identified for KS treatment by BSHS by importing the potential targets for KS and the targets for BSHS active ingredients (**Figure 2A**).

### 3.2 Construction of drug-compound-target networks

We used Cytoscape 3.7.2 software to construct the drug-compound-target network diagram. The purple circle nodes represented the 8 traditional Chinese medicines of BSHS, the hexagons nodes represented the compounds, the A1 and A2 nodes represented the common compounds of Lysimachiae Herba and Lygodii Spora, the A3 node represented the joint compound of Lysimachiae Herba and Licorice, the A4 node represented the joint compound of Lysimachiae Herba and Plantaginis Semen. The A5 and A6 nodes represented the common compounds of Licorice and Cibotii Rhizoma, the B1 node represented the joint compound of Lysimachiae Herba, Plantaginis Semen, and Licorice. The C1 node represented the joint compound of Lysimachiae Herba, Plantaginis Semen, Dipsaci Radix, and Licorice. The C2 node represented the joint compound of Malva verticillata seed, Dipsaci Radix, Lygodii Spora, and Cibotii Rhizoma. The D1 node represented the joint compound of Lysimachiae Herba, Lygodii Spora, Licorice, Clerodendranthus spicatus, and Cibotii Rhizoma, while the blue quadrilateral nodes represented the targets. The drug-compound-target network diagram included 365 nodes and 2713 edges (**Figure 2B**). The top five active compounds in BSHS, ranked according to the degree value, were quercetin, kaempferol, naringenin, β-sitosterol, and baicalein, which may play an essential role in treating kidney stones.

### 3.3 PPI network analysis and screening of key targets

In order to obtain the key proteins of BSHS in the treatment of KS, we constructed a PPI network with 239 nodes and 4557 edges based on a string database (**Figure 2C**). Based on the ‘degree’ value of topological parameters calculated by CytoNCA, 23 pivotal proteins were filtered out, including AKT1, IL6, MAPK3, TP53, VEGFA, CASP3, JUN, TNF, PTGS2, EGF, MAPK8, EGFR, STAT3, MYC, MMP9, MAPK1, ESR1, CXCL8, IL1β, CCND1, CAT, FOS, CCL2, which were strongly linked to KS (**Figures 2D, E**).

### 3.4 GO enrichment analysis and KEGG pathway analysis of key targets

We used the DAVID database to perform GO enrichment analysis of the 23 key targets for the identification of the relevant biological functions of BSHS against KS. The analysis uncovered 251 biological pathways, 22 cell localizations, and 35 molecular functions. As shown in **Figure 2F**, the top 10 terms in the biological process (BP), cellular component (CC), and molecular function (MF) categories that are significantly enriched are demonstrated. BP was found to be primarily associated with the negative regulation of apoptotic process, positive regulation of protein phosphorylation, lipopolysaccharide-mediated signaling pathway, and response to estradiol. The CC mainly included the extracellular space, protein complex, cytosol, nucleoplasm, etc. The MF mainly included enzyme binding, transcription factor binding, identical protein binding, etc.

In order to determine the potential pathway for BSHS in the treatment of KS, we performed a KEGG pathway enrichment analysis and found 102 signal pathways related to BSHS. A total of 20 pathways related to KS were screened, mainly including Tumour Necrosis Factor (TNF), estrogen, Mitogen-Activated Protein Kinase (MAPK), and Toll-like receptor signaling pathways. The enrichment pathway was visualized according to the size of the p-value (**Figure 2G**). In the pathways with the highest enrichment levels, estrogen and apoptosis signaling pathways were most closely related to KS. In addition, CAT is a key target of BSHS anti-kidney stones according to 2.4.3, which protects cells from oxidative stress by scavenging hydrogen peroxide produced by cellular metabolism (19). Multiple studies have also shown the harmful effects of oxidative stress on kidney stones (20, 21). Therefore, we predict that oxidative stress is also a key signaling pathway in the treatment of kidney stones by BSHS.

### 3.5 Experimental validation

#### 3.5.1 Identification and characterization of BSHS components

Total ion flow maps for the BSHS positive and negative ion scan modes were obtained from the data acquisition (**Supplementary Figures 2A, B**). The acquired data was processed with Xcailbur software. Matches were made in the HMDB and PubChem databases according to retention times, the mass information of the quasi-molecular and fragment ions, while keeping the quasi-molecular ions within ±5 ppm. A total of 52 compounds were eventually identified (**Supplementary Table 1**).

#### 3.5.2 BSHS inhibits the formation of calcium oxalate crystals

**Figure 3:**
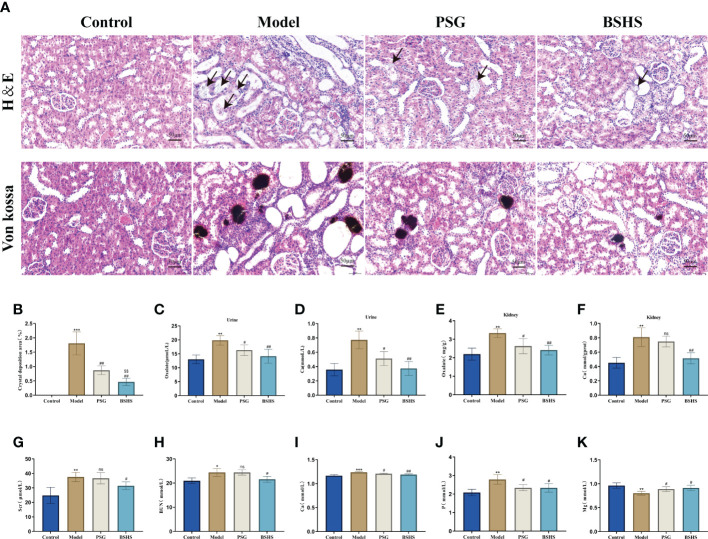
The therapeutic effect of BSHS on KS. **(A)** The H&E and Von Kossa staining of kidney sections from each group revealed tissue injury and CaOx crystal deposition. **(B)** The Image J (USA) software calculated the percentage of area positively stained for crystal deposition in each kidney section based on 20 random views at 200× magnification. **(C)** Oxalate content in the rat urine. **(D)** Ca content in the rat urine. **(E)** Oxalate content in the rat kidneys. **(F)** Ca content in the rat kidneys. **(G)** Cr content in the rat serum. **(H)** Blood urea nitrogen (BUN) content in the rat serum. **(I)** Ca content in the rat serum. **(J)** P content in the rat serum. **(K)** Mg content in the rat serum. All values were expressed as mean ± SD. ^*^p<0.05 *vs*. the normal control group, ^**^p < 0.01 *vs*. the normal control group, ^***^p  < 0.001 *vs*. the normal control group, *^#^p *< 0.05 *vs*. the model group, *^##^p* < 0.01 *vs*. the model group, *^$$^p* < 0.01 *vs*. the PSG group, ns for *p*>0.05 *vs*. the model group.

We investigated the impact of BSHS on renal injury and calcium oxalate crystal deposition after EG+AC induction *in vivo*. H&E and Von Kossa staining of kidney sections revealed renal tubule severe dilation, tubule destruction, and epithelial cell desquamation induced by EG+AC. A large amount of calcium oxalate crystal deposition was noticed after EG+AC induction, whereas coadministration of BSHS could protect the EG+AC-injured kidney tissue from inflammatory damage and calcium oxalate crystal deposition (**Figures 3A, B**).

#### 3.5.3 BSHS increases 24 h urine volume and improves renal/body weight index in rats

On day 28, we recorded 24h urine volume and weighed the body weight and renal weight of rats. The results showed that there were significantly decreased 24 h urine volume and increased renal/body weight index in the model group rats compared to the normal control group. Simultaneous treatment with BSHS resulted in a remarkable decrease in renal/body weight index, as well as an increase in 24 h urine volume in the rat (**Supplementary Figures 3A, B**).

#### 3.5.4 BSHS regulated urine and renal biological parameters in rats

In this study, we investigated whether BSHS inhibited the formation of oxalate, Ca, and P in an animal model, and increased the level of Mg. As expected, there was a significant increase (p<0.05) of oxalate and calcium contents in both the kidneys and urine of rats in the model group compared to those in the normal control group. Simultaneous treatment with BSHS resulted in a significant decrease (p<0.05) in oxalate and calcium levels in the kidney of rats (**Figures 3C–F**). BSHS also decreased the level of P and increased the level of Mg in the urine of rats compared to the model group (**Supplementary Figures 3C, D**).

#### 3.5.5 BSHS regulated serum biological parameters in rats

Meanwhile, to examine the effect of BSHS on the renal function and serum biological parameters of rats, we further examined the levels of Cr, BUN, Ca, P, and Mg in the serum of rats. The results showed that there were significantly decreased Mg content and increased Ca, P, Cr, and BUN contents in the serum of model group rats compared to the normal control group. Simultaneous treatment with BSHS resulted in a remarkable decrease in Ca, P, Mg, Cr, and BUN levels, as well as an increase in Mg levels in the rat serum. Worthy of note, there was no significant trend of lowering Cr and BUN in the PSG group compared to the model group, suggesting a better effect of BSHS in improving renal function (**Figures 3G–K**).

#### 3.5.6 BSHS inhibited apoptosis induced by EG+AC in rat

**Figure 4:**
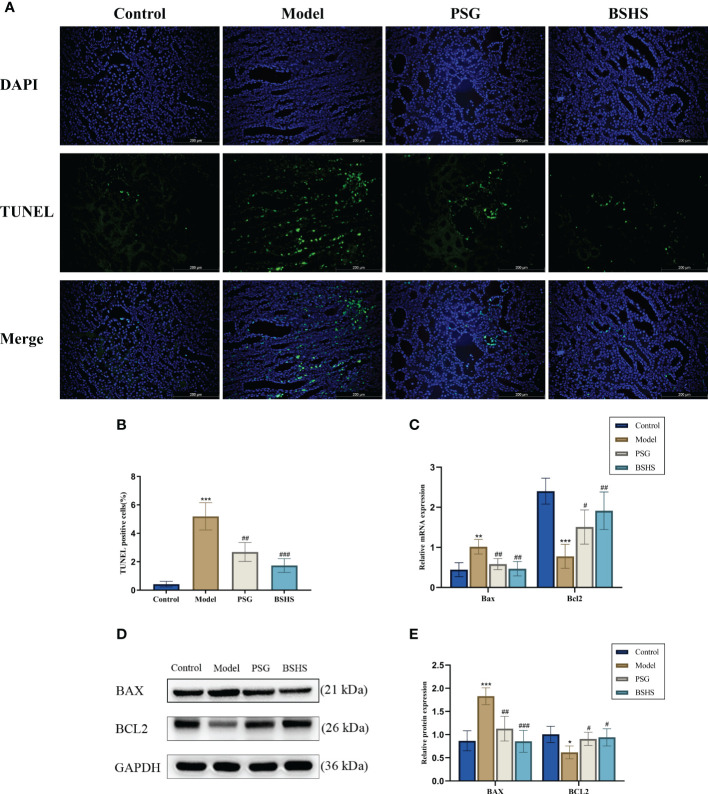
Effect of BSHS on EG+AC-induced apoptosis in rat kidney tissue. **(A)** TUNEL staining was used to assess renal apoptosis. **(B)** Images J was used to count the percentages of TUNEL-positive cells (green) to total cells (blue). **(C)** The expression of apoptosis-related genes was evaluated by qRT-PCR. **(D)** The expression of apoptosis-related proteins was evaluated by Western bolting. **(E)** A graph showing the semi-quantitative analysis of BAX and BCL2. Data are presented as the mean ± SD and density normalized to GAPDH. ^*^ *p*<0.05 *vs*. the normal control group, ^**^ *p *< 0.01 *vs*. the normal control group, ^***^ *p* < 0.001 vs. the normal control group, ^#^ *p* < 0.05 *vs*. the model group, ^##^ *p* < 0.01 *vs*. the model group, ^###^ *p* < 0.001 *vs*. the model group.

Network pharmacological analysis indicated that apoptosis might be involved in BSHS treatment of kidney stones. Several studies have also demonstrated that apoptosis is crucial in kidney stone formation (22–24). Therefore, our study examined the apoptotic effects of BSHS on KS in animal models. In the model group, TUNEL-positive cells were significantly higher than in the normal control group, according to the TUNEL staining results. Contrary to what was observed in the model group, BSHS groups showed fewer apoptotic cells (**Figures 4A, B**). The qRT-PCR results indicated that *Bax* levels were significantly increased and *Bcl2* was decreased in the model group, while treatment with BSHS reversed the increase of *Bax* levels and restored *Bcl2* expression (**Figure 4C**). The Western bolting results indicated that BAX and Cleaved Caspase-3/Caspase-3 levels were significantly increased and BCL2 was decreased in the model group, while treatment with BSHS reversed the increase of BAX and Cleaved Caspase-3/Caspase-3 levels and restored BCL2 expression (**Figures 4D, E**) (**Supplementary Figures 4A, B**).

#### 3.5.7 BSHS improves the imbalance of estrogen levels induced by EG+AC in rat

**Figure 5:**
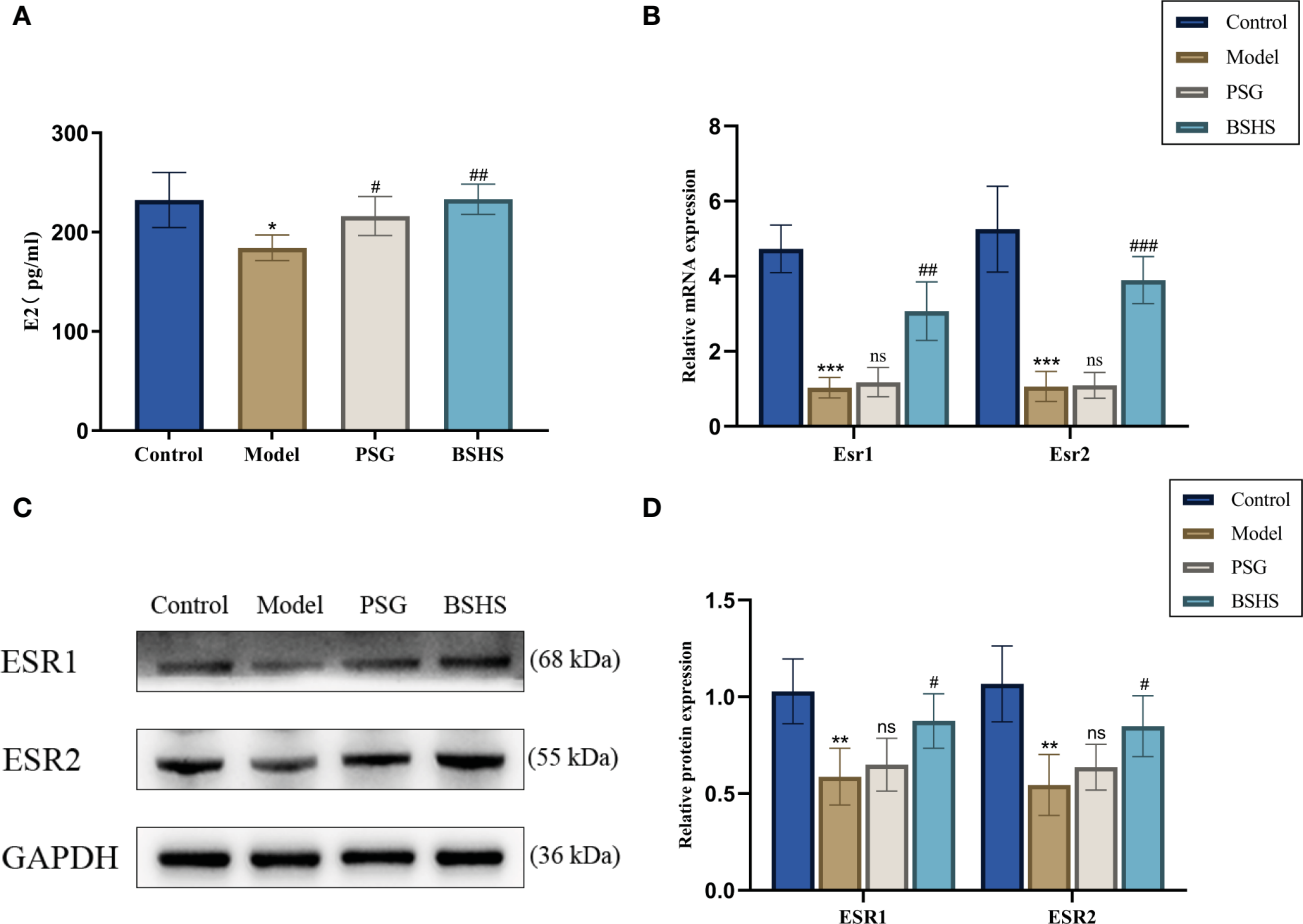
The effect of BSHS on the imbalance of estrogen levels induced by EG+AC in the rat. **(A)** ELISA evaluated the expression of estrogen levels. **(B)** The expression of estrogen receptor-related genes was evaluated by qRT-PCR. **(C)** The expression of estrogen receptor-related proteins was examined by Western blotting. **(D)** A semi-quantitative analysis of ESR1 and ESR2 is shown. Data are presented as the mean ± SD and density normalized to GAPDH. ^*^ *p*<0.05 *vs*. the normal control group, ^**^ *p* < 0.01 *vs*. the normal control group, ^***^ *p* < 0.001 *vs*. the normal control group, ^#^ *p* < 0.05 *vs*. the model group, ^##^ *p* < 0.01 *vs*. the model group, ^###^ *p<* 0.001 *vs*. the model group, ns for *p* > 0.05 *vs*. the model group.

Network pharmacological analysis suggested estrogen signaling pathways may be involved in BSHS treating kidney stones. Interestingly, lower estrogen levels have also been shown to be strongly associated with the formation of kidney stones (25–27). As part of our study, we examined the effect of BSHS on estrogen and estrogen receptors in this animal model. E2 serum levels were found to be lower in the model group compared with the control group in the ELISA experiment. In contrast, it more dramatically increased in the BSHS groups than in the model group (**Figure 5A**). The qRT-PCR results illustrated that *Esr1* and *Esr2* mRNA levels were remarkably decreased in the model group, while treatment with BSHS reversed the decrease in *Esr1* and *Esr2* levels (**Figure 5B**). The Western blotting results showed the same trend (**Figures 5C, D**).

#### 3.5.8 BSHS alleviated EG+AC-induced oxidative stress in rats

**Figure 6:**
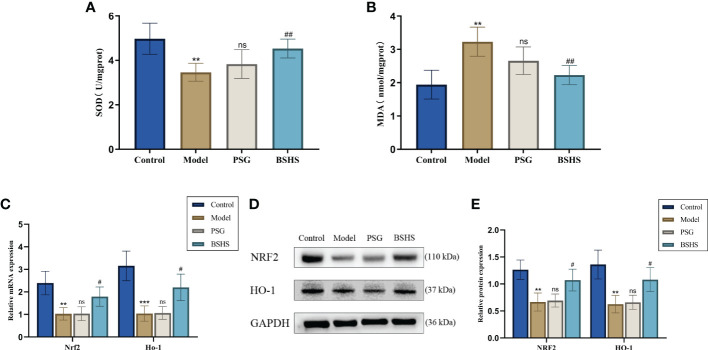
A comparison of oxidative stress levels in kidneys among different groups. **(A)** SOD activity in the kidneys of rats. **(B)** MDA content in the kidneys of rats. **(C)** qRT-PCR was used to evaluate the expression of genes related to oxidative stress. **(D)** The expression of oxidative stress-related proteins was evaluated by Western blotting. **(E)** A semi-quantitative analysis of NRF2 and HO-1 is shown. Data are presented as the mean ± SD and density normalized to GAPDH. ^**^ *p* < 0.01 *vs*. the normal control group, ^***^ *p *< 0.001 *vs*. the normal control group, ^#^ *p* < 0.05 *vs*. the model group, ^##^ *p* < 0.01 *vs*. the model group, ns for *p* > 0.05 *vs*. the model group.

According to growing evidence, oxidative stress may play an essential role in hyperoxaluria-induced kidney injury, resulting in renal CaOx crystallization (28–30). Herein, we examined the potential antioxidative properties of BSHS in this animal model. As expected, compared to the normal control group, rats in the model group significantly increased MDA content and decreased SOD activity in the kidneys. The simultaneous treatment of rats with BSHS reduced MDA levels in the kidneys and increased SOD activity (**Figures 6A, B**). The qRT-PCR results illustrated that *Nrf2* and *Ho*-*1* mRNA levels were remarkably decreased in the model group, while treatment with BSHS reversed the decrease in *Nrf2* and *Ho*-*1* levels (**Figure 6C**). The Western blotting results showed a similar trend (**Figures 6D, E**). Interestingly, the PSG group did not show significant antioxidant effects, while the BSHS group had great antioxidant capacity.

## 4 Discussion

Kidney stones are a common and frequently-occurring disease of the urinary system, and their incidence increases annually (31). According to the most recent epidemiological study conducted in China, kidney stones are prevalent in approximately 5.8% of the population (32). It is estimated that 12% of men and 6% of women in the world population will have kidney stones at least once in their lifetime, with recurrence rates of 70–80% for men and 47–60% for women (33). Among them, calcium oxalate stones are the most common kidney stones (34, 35), accounting for over 80% of them (36). Although people have an in-depth understanding of crystallization and stone formation, there is currently a lack of effective treatment methods and drugs due to the slow progress in determining the pathophysiology of stone formation. Therefore, kidney stone disease must be given sufficient attention. The expansion of the treatment model of kidney stone disease based on TCM can provide a reliable solution for the pathogenesis of kidney stone disease that is difficult to cure and easy to relapse. Although under the guidance of the holistic view of TCM, Chinese herbal compound has an excellent therapeutic effect on diseases. Due to their complex components, multi-target, and multi-channel treatment characteristics, it is not easily accessible for an in-depth study of its internal mechanism. In recent years, network pharmacology has become a popular technique for analyzing the mechanism of action of complex TCM prescriptions (37). The combination of network pharmacology and experimental verification was used in this study in order to clarify the pharmacological mechanism of BSHS against kidney stones.

It is well known that the formation of kidney stones is a complex process involving urinary supersaturation, nucleation, growth, aggregation, and retention of urinary stone components within the renal tubular cells (38). Multiple studies have shown that kidney stone formation could be attributed to higher supersaturation of urine because of low urine volume and increased secretion of calcium, phosphates, oxalates, uric acid, and cysteine in urine (39–41). The elevated urinary excretion of calcium (hypercalciuria) and oxalate (hyperoxaluria) are the most common risk factor for CaOx kidney stones (42, 43). However, some scholars supported that oxalate in the urine combines with free calcium to form insoluble CaOx, which induces kidney stones and can lead to a decrease in urinary calcium (44, 45). Interestingly, we discovered that BSHS can decrease urine oxalates and calcium excretion which may be related to the fact that BSHS increase urinary magnesium levels. Studies have shown that magnesium can compete with calcium to bind oxalate and form insoluble solutes that are excreted in the urine (46). Low urinary oxalate concentrations lead to a reduction in urinary calcium levels, therefore BSHS can treat kidney stones by reducing urinary oxalate, urinary calcium and increasing urinary magnesium levels.

TCM compounds that lacked proper pharmacokinetic properties would not reach their target organs to exert their biological effects (47). It has been demonstrated that compounds with OB ≥30% and DL index ≥0.18 may be absorbed and distributed in the human body and are thus considered pharmacokinetically active (48, 49). Compounds with high-degree may explain the significant therapeutic effects of BSHS on kidney stones in the compound-key targets network. According to this study, quercetin was the most significant compound, followed by kaempferol, naringenin, β-sitosterol, and baicalein. It is reported that quercetin, a natural flavonoid, has efficient antioxidant properties and can be used to inhibit oxidative damage in renal tubular cells and tissues (50). In addition, quercetin can inhibit the formation of urinary tract stones induced by oxalate (51). Kaempferol is one of the most common glycoside forms of aglycon flavonoids, which can increase the level of coenzyme Q in kidney cells to play an antioxidant role (52). As a naturally occurring flavanone, naringenin inhibits oxidative stress in the kidneys and improves kidney function (53). β-Sitosterol is a phytosterol reported in ancient medicinal history for treating nephritis and prostatitis (54). β-sitosterol has been reported to inhibit nephrotoxicity and anti-kidney oxidation properties (55, 56). Baicalein is a member of the flavonoid family, and modern pharmacology proves that baicalein can inhibit inflammation by activating the Nrf2 signaling pathway, thereby alleviating lupus nephritis (57). Oxidative stress-induced apoptosis of renal tubular epithelial cells is a risk factor for stone formation (20). All these works demonstrate that BSHS has excellent anti-kidney oxidation and renal protection.

It has been revealed that BSHS acts on multiple targets using multiple signaling pathways when we integrate the topological network parameters with all the network analyses. We finally identified estrogen, apoptosis, and oxidative stress as crucial mechanisms for BSHS treatment of kidney stones based on network pharmacology analysis. Multiple clinical studies have suggested that estrogen has a protective effect during the formation of kidney stones (58, 59). However, the Women’s Health Initiative Study and the Nurses’ Health Study found no positive correlation between hormone replacement therapy and the prevention of kidney stones (59, 60). These results have caused scholars to question the relationship between estrogen and kidney stones. For this result, some researchers suggested that the long-term estrogen decline caused by menopause may aggravate the deterioration of normal physiological estrogen receptor function in the kidney (60). Therefore, the poor effect of hormone replacement therapy on renal calculi may be due to the reduced protein expression of estrogen receptors or its cofactors in these women (60). We validated the effects of BSHS on E2 and estrogen receptors *in vivo*. The results showed that BSHS could not only increase the level of E2 but also increase the levels of ESR1 and ESR2. There are growing numbers of studies demonstrating that the adhesion or endocytosis of renal tubular epithelial cells to crystals plays an essential role in forming stones (61, 62). Moreover, crystal adhesion can be enhanced by injured renal tubular epithelial cells, which can promote kidney stones (34). Interestingly, the damage to renal tubular epithelial cells is closely related to oxidative stress (63). As an essential antioxidant pathway for endogenous anti-oxidative stress in cells (64, 65), the NRF2/HO-1 signaling pathway is vital in improving oxidative stress in kidney diseases (66–68). Many studies have confirmed that the inhibitory effect of the estrogen signaling pathway on oxidative stress is closely related to the activation of the NRF2/HO-1 antioxidant pathway (69, 70). Interestingly, our *in vivo* studies showed that BSHS could not only increase the expression of NRF2 and HO-1 proteins and genes but also increase SOD activity and decrease MDA levels in the rat kidney. It is suggested that BSHS has a good anti-oxidative stress effect on the kidney. It has been reported that renal tubular epithelial cell apoptosis is an essential factor that causes crystals to adhere to renal tubular epithelial cells (22). Some scholars suggested that oxidative stress is a risk factor for apoptosis (71). BCL2/BAX signaling pathway is a pivotal way to regulate cell apoptosis. Our studies demonstrated that BSHS increases the expression of BCL2 and reduces the expression of BAX, thereby reducing the level of apoptosis of renal tubular epithelial cells. Therefore, the therapeutic effect of BSHS on the calcium oxalate stone model in rats may be related to the increase of estrogen receptor levels and the inhibition of apoptosis.

Our findings suggest that BSHS may inhibit kidney stone formation mainly by regulating estrogen and estrogen receptor levels, inhibiting oxidative stress processes, reversing apoptosis, and decreasing CaOx crystals deposition through E2/ESR1/ESR2, NRF2/HO-1, and BCL2/BAX signaling pathways. Overall, BSHS ameliorated KS progression through a multi-ingredient, multi-target, and multi-pathway mode, which is different from chemical drugs that work on a distinct and single target. The understanding of complex interactions between disease and chemical ingredients in TCM could be well accomplished by identifying network targets and signaling pathways. It is important to note, however, that this study has some limitations. First, the results may have been slightly skewed since we only validated part of the core pathways and targets of BSHS. Therefore, further validation of other relevant targets and signaling pathways predicted by network pharmacology would be required in future experiments. Secondly, our study did not demonstrate an association between estrogen, oxidative stress, and apoptotic signaling pathways. In a follow-up experiment, we will examine their connection through *in-vitro* experiments.

## 5 Conclusions

In summary, network pharmacology analysis coupled with experimental validation was performed to decipher the molecular mechanisms of BSHS in the treatment of KS. The network pharmacology analysis revealed that BSHS exerted anti-KS effects *via* multi-ingredients, multi-targets, and multi-pathways. The experimental results verified that BSHS improved CaOx crystal deposition in KS by modulating the E2/ESR1/ESR2, NRF2/HO-1, and BCL2/BAX signaling pathways. This study could provide an optimized method to elucidate the pharmacological mechanisms of BSHS and supply a novel candidate for treating KS.

## Data availability statement

The original contributions presented in the study are included in the article/**Supplementary Material**. Further inquiries can be directed to the corresponding authors.

## Ethics statement

The animal study was reviewed and approved by the experimental animal ethics committee of the Tianjin University of Traditional Chinese Medicine.

## Author contributions

YB and YW conceived this project. HL and MC designed the study, wrote the manuscript, and performed the experiments. YJ performed the network pharmacology and data analysis. BJ, MD, and LH edited the manuscript. LW performed the UHPLC-Q/Orbitrap MS experiments. JA, JL, and TZ revision of the manuscript. BC provides drug prescriptions. All authors contributed to the article and approved the submitted version.

## Supplementary material

Supplementary Figure 1 Drug composition of BSHS.

Supplementary Figure 2 BSHS total ion flow diagram. **(A)** negative; **(B)** positive.

Supplementary Figure 3 The therapeutic effect of BSHS on KS. **(A)** 24 h urine volume. **(B)** Renal/body weight index. **(C)** Mg content in the rat urine. **(D)** P content in the rat urine. All values were expressed as mean ± SD. *^*^p*<0.05 *vs*. the normal control group, *^**^p*<0.01 *vs*. the normal control group, *^***^p*<0.001 *vs*. the normal control group, *^#^p*<0.05 *vs*. the model group, *^##^p*<0.01 *vs*. the model group, ns for *p*>0.05 *vs*. the model group.

Supplementary Figure 4 Effect of BSHS on EG+AC-induced apoptosis in rat kidney tissue. **(A)** The expression of Caspase-3 and Cleaved Caspase-3 proteins was evaluated by Western bolting. **(B)** A graph showing the semi-quantitative analysis of Cleaved Caspase-3/Caspase-3. Data are presented as the mean ± SD and density normalized to GAPDH. ^**^ *p*<0.01 *vs*. the normal control group, ^#^ *p*<0.05 *vs*. the model group, ^##^ *p*<0.01 *vs*. the model group.
