# Supplementary figures and images for: Network pharmacology and experimental validation to elucidate the pharmacological mechanisms of Bushen Huashi decoction against kidney stones
Source: Front Endocrinol (Lausanne). 2023 Feb 14;14:1031895. doi: 10.3389/fendo.2023.1031895 (PMC9971497; doi:10.3389/fendo.2023.1031895)

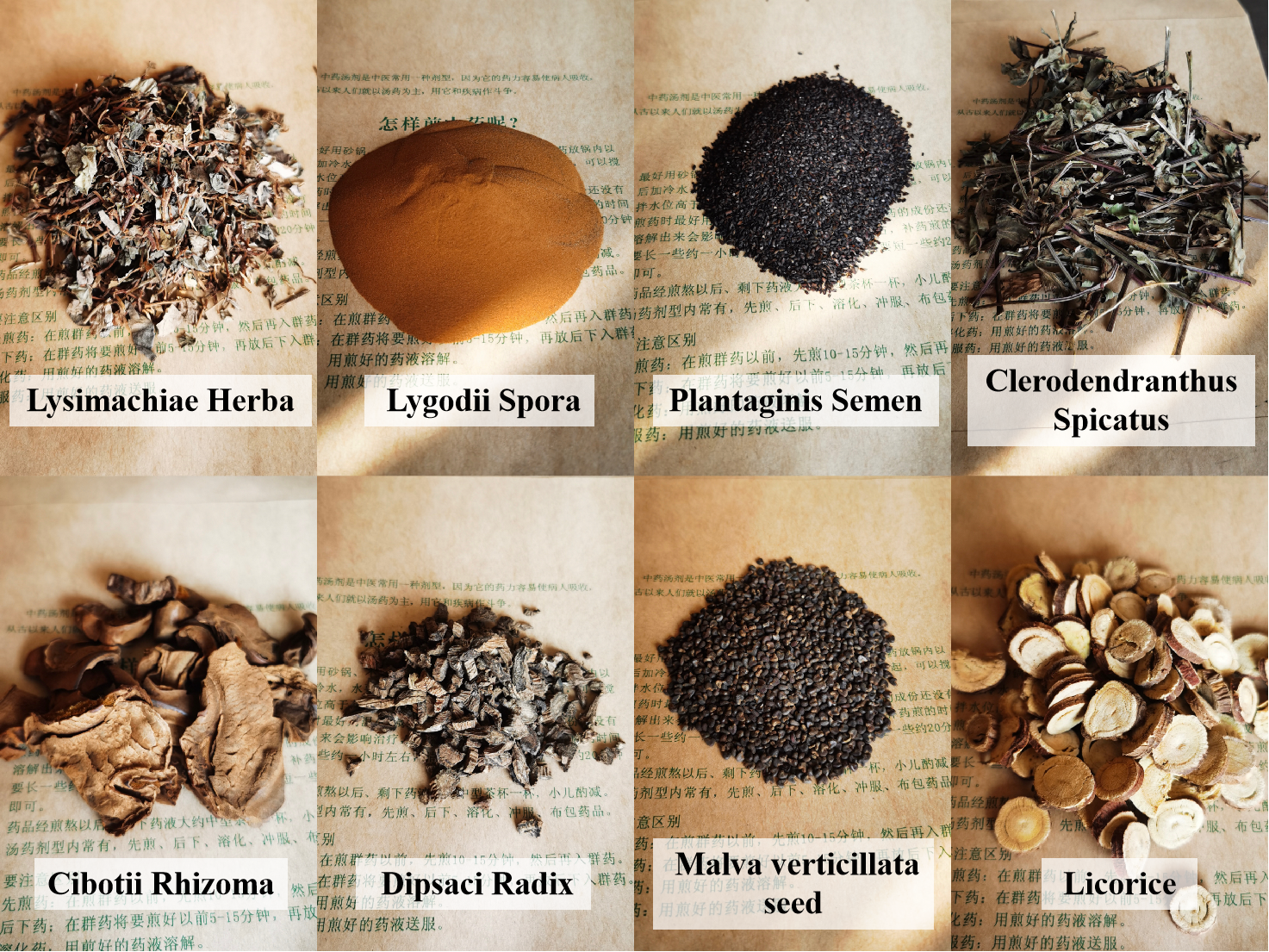

Supplement: Supplementary Figure 1 — Drug composition of BSHS. [file Image_1.tif]

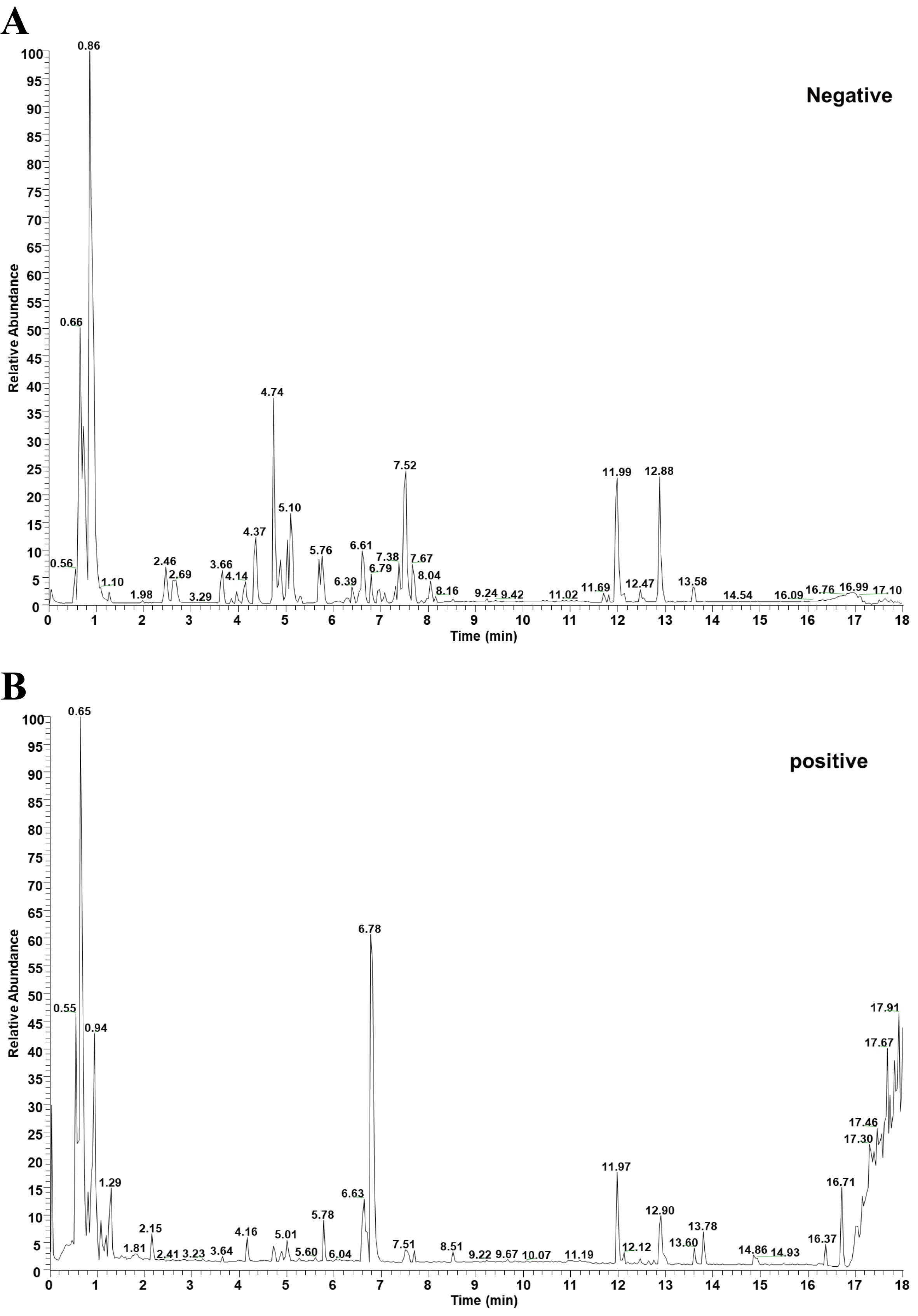

Supplement: Supplementary Figure 2 — BSHS total ion flow diagram. (A) negative; (B) positive. [file Image_2.tif]

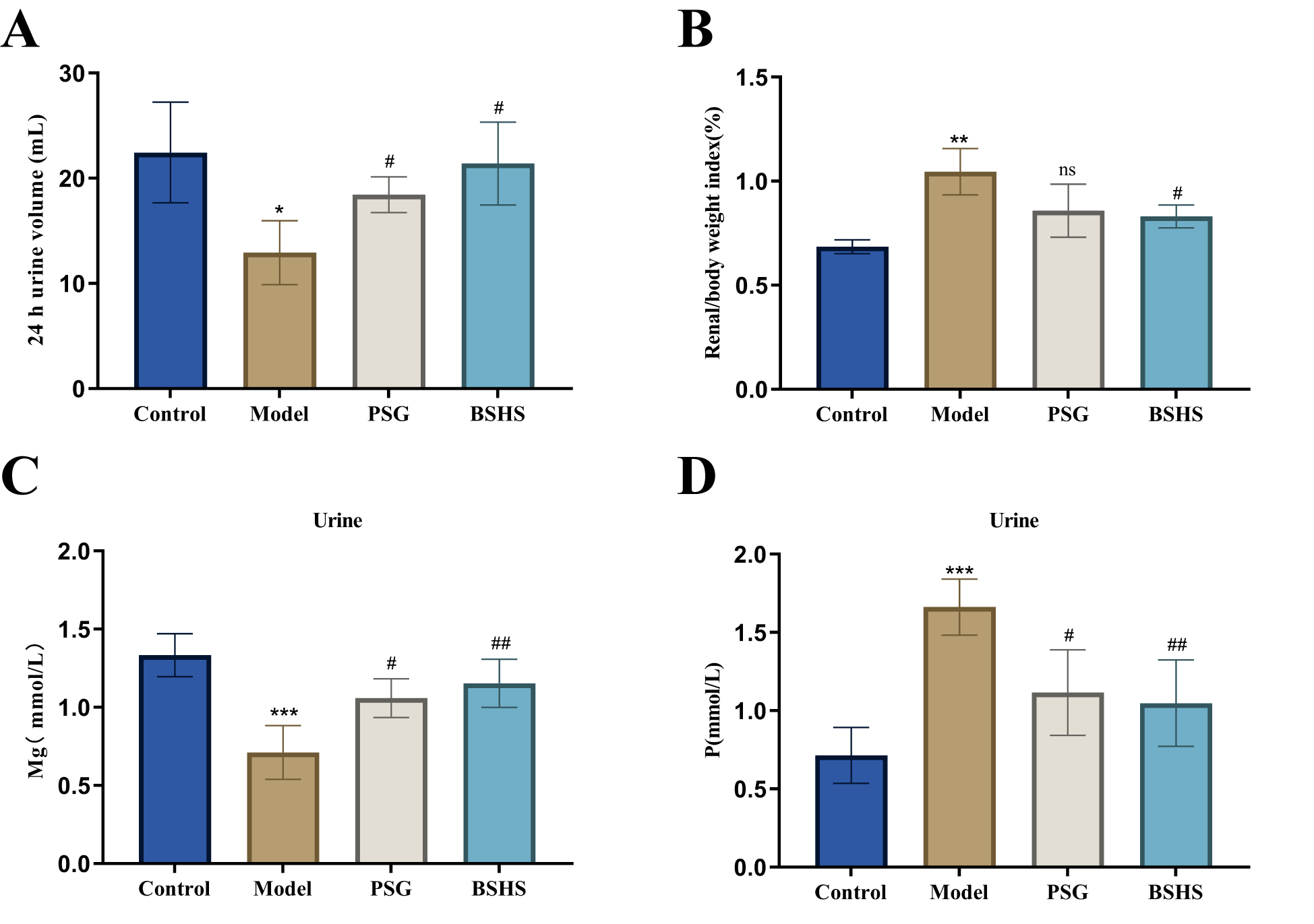

Supplement: Supplementary Figure 3 — The therapeutic effect of BSHS on KS. (A) 24 h urine volume. (B) Renal/body weight index. (C) Mg content in the rat urine. (D) P content in the rat urine. All values were expressed as mean ± SD. *p<0.05 vs. the normal control group, **p<0.01 vs. the normal control group, ***p<0.001 vs. the normal control group, #p<0.05 vs. the model group, ##p<0.01 vs. the model group, ns for p>0.05 vs. the model group. [file Image_3.tif]

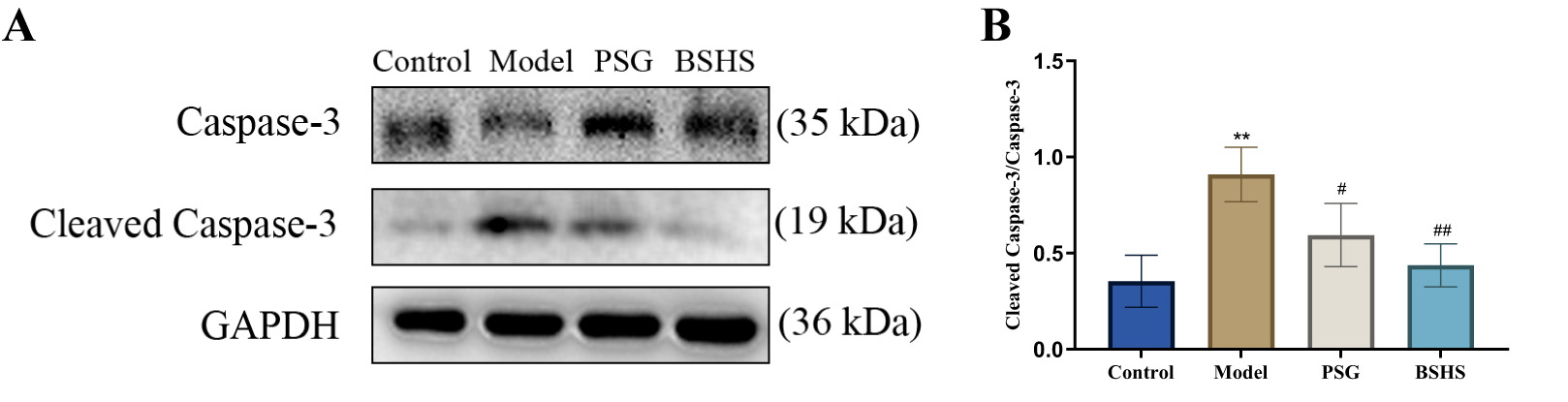

Supplement: Supplementary Figure 4 — Effect of BSHS on EG+AC-induced apoptosis in rat kidney tissue. (A) The expression of Caspase-3 and Cleaved Caspase-3 proteins was evaluated by Western bolting. (B) A graph showing the semi-quantitative analysis of Cleaved Caspase-3/Caspase-3. Data are presented as the mean ± SD and density normalized to GAPDH. ** p<0.01 vs. the normal control group, # p<0.05 vs. the model group, ## p<0.01 vs. the model group. [file Image_4.tif]
