# Supplementary material for: Network pharmacology and experimental validation to elucidate the pharmacological mechanisms of Bushen Huashi decoction against kidney stones
Source: Front Endocrinol (Lausanne). 2023 Feb 14;14:1031895. doi: 10.3389/fendo.2023.1031895 (PMC9971497; doi:10.3389/fendo.2023.1031895)
Supplement: Supplementary file 5 [file Table_1.docx]

**Supplementary Table 1 Identification of the main compounds of BSHS based on UHPLC-Q/Orbitrap MS**

| **NO.** | **Compound** | **Formula** | **RT** | ***m/z*** | **Adduct** | **MS/MS** |
| --- | --- | --- | --- | --- | --- | --- |
| 1 | Isorhamnetin | C16H12O7 | 1.12 | 317.06293 | [M+H]^+^ | 283.42599,225.37787,179.44258,124.08964,70.06580 |
| 2 | Protocatechuic acid | C7H6O4 | 2.61 | 153.01840 | [M-H]^-^ | 141.91104，123.90018，116.69644，109.02786，103.91908，98.84242，82.02832，71.42588，61.98690，53.70065 |
| 3 | Geniposidic acid | C16H22O10 | 3.66 | 373.11359 | [M-H]- | 193.04971,179.05502,149.05959,97.02809,59.01249 |
| 4 | Chlorogenic acid | C16H18O9 | 4.90 | 355.10240 | [M+H]^+^ | 163.03879,145.02829,135.04396,117.03365,89.03899 |
| 5 | Caffeic acid | C9H8O4 | 5.10 | 179.03398 | [M-H]- | 150.95280，144.87024，135.04382，130.15881，122.89273，113.02293，104.92671，101.02260，90.99677，85.02790，71.01244，59.01249，57.03320 |
| 6 | Quercetin | C15H10O7 | 6.09 | 303.04904 | [M+H]^+^ | 279.19507,226.89279,210.29794,185.09830,142.08557,120.04482,110.06045,99.86438,84.96049,69.07052,55.68917 |
| 7 | Kaempferol | C15H10O6 | 6.27 | 287.05481 | [M+H]^+^ | 258.05234,213.05447,153.01817,121.02857 |
| 8 | Rutin | C27H30O16 | 6.47 | 609.14581 | [M-H]- | 328.68106,255.02950,243.02943,227.03473,191.05539178.88754,151.00247,107.01228,65.00181 |
| 9 | Ferulic acid | C10H10O4 | 6.47 | 193.04974 | [M-H]- | 178.02615,149.05957,137.02313,134.03595,61.98692 |
| 10 | Isoquercitrin | C21H20O12 | 6.51 | 463.08795 | [M-H]- | 316.02213,287.01923,271.02469,242.02155,151.00259,119.04900 |
| 11 | Hypericin | C30H16O8 | 6.61 | 503.07724 | [M-H]- |  |
| 12 | Liquiritin | C21H22O9 | 6.76 | 417.11938 | [M-H]- | 255.06606,153.01819,135.00743,119.04887,91.01745 |
| 13 | Luteolin | C15H10O6 | 7.15 | 287.05502 | [M+H]^+^ | 231.06462,216.48590,164.74626,153.01859,143.26031,131.97418,113.96369,101.52341,85.34367,72.93755,57.07080 |
| 14 | Naringenin | C15H12O5 | 7.41 | 273.07553 | [M+H]^+^ | 267.70847,184.63000,171.02869,153.01817,147.04369,119.04934,107.0493591.05470,67.01848 |
| 15 | Isoliquiritin | C21H22O9 | 8.13 | 417.11871 | [M-H]- | 255.06595,180.00534,148.01530,135.00735,108.02022,91.01744 |
| 16 | Liquiritigenin | C15H12O4 | 8.70 | 257.08063 | [M+H]^+^ | 239.06981,211.07472,147.04391,137.02327,119.04933 |
| 17 | Methyl rosmarinate | C19H18O8 | 9.10 | 373.09289 | [M-H]- | 197.04466,175.03896,160.01535,135.04379,72.99165 |
| 18 | Baicalein | C15H10O5 | 11.48 | 271.06110 | [M+H]^+^ | 151.00243,119.04881,107.01224,65.00180 |
| 19 | Akebia saponin D | C47H76O18 | 11.97 | 929.50990 | [M+H]^+^ | 789.76428,455.35156,437.34207,409.34573,189.16393 |
| 20 | Isoliquiritigenin | C15H12O4 | 12.26 | 255.06602 | [M-H]- | 219.84471,213.05403,184.87511,153.01813,145.02847,135.00743,119.04884,91.01746 |
| 21 | Glycyrrhizic acid | C42H62O16 | 12.85 | 821.39600 | [M-H]- | 351.05652,193.03448,113.02302,85.02805 |
| 22 | Corosolic acid | C30H48O4 | 13.61 | 471.34650 | [M-H]- | 453.33585,435.32419,317.21191,189.16359,95.08601 |
| 23 | Lico-iso-flavone B | C20H16O6 | 14.58 | 351.08719 | [M-H]- | 335.05612,321.03961,203.03461,107.01215 |
| 24 | Licoisoflavanone | C20H18O6 | 15.65 | 353.10322 | [M-H]- |  |
| 25 | Trigonelline | C7H7NO2 | 0.655 | 138.0553 | [M+H]^+^ | 110.06016,103.05466,95.04953,93.07037,91.05470,81.07050,56.96560 |
| 26 | Vigabatrin | C6H11NO2 | 1.819 | 130.08625 | [M+H]^+^ | 113.96392,96.00970,87.00452,71.04980,70.06579 |
| 27 | Tuliposide A | C11H18O8 | 11.709 | 277.09256 | [M-H]- | 119.03352,101.02296,97.02802,89.02293,85.02798,71.01237 |
| 28 | 3,4-Difluorocinnamic acid | C9H6F2O2 | 1.276 | 183.02849 | [M-H]- | 155.91928,138.90540,119.26656,103.91895,96.95869,61.98687 |
| 29 | Gluconic acid δ-lactone | C6H10O6 | 0.957 | 177.03944 | [M-H]- | 159.02875,115.03869 |
| 30 | trans-3-Indoleacrylic acid | C11H9NO2 | 4.296 | 188.07063 | [M+H]^+^ | 170.06003,144.08083,142.06511,115.05450,91.05477 |
| 31 | Vanillyl mandelic acid | C9H10O5 | 7.508 | 197.04472 | [M-H]- | 179.03401,162.83797,135.04381,123.04379,72.99171 |
| 32 | Trehalose | C12H22O11 | 0.654 | 341.10893 | [M-H]- | 179.05525,113.02307,101.02302,89.02298,71.01238,59.01244 |
| 33 | Traumatic Acid | C12H20O4 | 12.359 | 227.12825 | [M-H]- | 180.83002,165.12744,152.91714,143.86369,99.92429,61.98687 |
| 34 | Uridine | C9H12N2O6 | 0.948 | 243.06195 | [M-H]- | 200.05577,128.03394,115.00220,110.02335,82.02838 |
| 35 | Zeatin | C10H13N5O | 2.936 | 220.11789 | [M+H]^+^ | 202.10703,174.11298,128.95132,113.96410,97.00830,90.09171 |
| 36 | Viloxazine | C13H19NO3 | 2.378 | 238.14351 | [M+H]^+^ | 220.10788,152.10692,134.09634,121.08865,87.04454 |
| 37 | Fraxetin | C10H8O5 | 4.56 | 207.02919 | [M-H]- | 179.93562,163.03891,159.85886,145.02802,135.04353,127.86869,118.99167,103.91875,87.92383,73.02804,61.98693 |
| 38 | D-Malic acid | C4H6O5 | 0.7 | 133.01291 | [M-H]- | 115.00230，89.02300，73.99498，72.99169，71.01240 |
| 39 | D-Quinic acid | C7H12O6 | 4.89 | 191.05518 | [M-H]- | 173.04466，146.93730，127.03864，102.94737，87.92379，85.02805 |
| 40 | D-Glucose | C6H12O6 | 0.7 | 179.05521 | [M-H]- | 161.04437，143.03391，131.03377，101.02302，89.02297，75.00732，71.01239，59.01243 |
| 41 | 2-Furoic acid | C5H4O3 | 16.9 | 113.02364 | [M+H]^+^ | 114.09171,112.08727,96.08121,83.06092,70.06577 |
| 42 | Tangeritin | C20H20O7 | 13.09 | 373.12817 | [M+H]^+^ | 357.09723,343.08066,327.08685,312.09995,193.39856,153.01794,119.70806,80.85575 |
| 43 | Suberic acid | C8H14O4 | 6.42 | 173.08139 | [M-H]- | 154.94630,143.86348,128.87674,117.61189,115.91933,111.08009,107.11842,99.92439,61.98692 |
| 44 | Rubone | C20H22O7 | 13.16 | 375.14322 | [M+H]^+^ | 298.23810，238.93718，211.05994，196.03648，183.02875，167.03358150.03131，122.03645，94.04177，66.04718 |
| 45 | piscidic acid | C11H12O7 | 2.8 | 255.05077 | [M-H]- | 219.84456，193.04971，179.03398，165.05464，149.05966，133.02814，107.04883，93.03322，72.99168，58.00449 |
| 46 | Neochlorogenic acid | C16H18O9 | 2.9 | 355.10172 | [M+H]^+^ | 285.00888，267.31961，230.91525，211.47772，91.05793，57.65622 |
| 47 | L-Threonic acid | C4H8O5 | 0.64 | 135.0285 | [M-H]- | 117.01799,99.00740,89.02293,76.01067,75.00730,72.99165,61.98691,59.01242,55.01756 |
| 48 | Kaempferol 3-robinobioside | C27H30O15 | 6.55 | 593.15076 | [M-H]- | 284.03235,255.02948,227.03441,211.03918,151.00262,107.01229,61.98689 |
| 49 | Kaempferide 3,7-dirhamnoside | C28H32O14 | 8.05 | 591.17157 | [M-H]- | 531.69000,463.17780,255.06590,153.01787,135.00742,119.04875,110.53234,91.01733,59.01232 |
| 50 | Indole-3-acetic acid | C10H9NO2 | 7.04 | 176.07047 | [M+H]^+^ | 159.04340,148.07567,130.06522,120.08095,109.30595,95.70505,91.05459,70.06570,62.93915 |
| 51 | Hesperetin | C16H14O6 | 7.46 | 301.0722 | [M-H]- | 286.04764,269.04535,527.08093,191.03389,177.01770,161.02335,150.03107,139.30197,124.01521,109.02820,90.70943,67.65874 |
| 52 | Gallic acid | C7H6O5 | 1.28 | 169.01315 | [M-H]- | 162.44907,155.62967,149.03131,125.02306,122.89288,107.01247,97.02808,81.03326,69.03308,61.98680 |
